# Case management for frequent users of the emergency department: study protocol of a randomised controlled trial

**DOI:** 10.1186/1472-6963-14-264

**Published:** 2014-06-17

**Authors:** Patrick Bodenmann, Venetia-Sofia Velonaki, Ornella Ruggeri, Olivier Hugli, Bernard Burnand, Jean-Blaise Wasserfallen, Karine Moschetti, Katia Iglesias, Stéphanie Baggio, Jean-Bernard Daeppen

**Affiliations:** 1 Department of Ambulatory Care and Community Medicine, University of Lausanne, Lausanne CH-1015, Switzerland; 2 Department of Community Medicine and Public Health, Lausanne University Hospital, Lausanne CH-1011, Switzerland; 3 Emergency Department, Lausanne University Hospital, Lausanne CH-1011, Switzerland; 4 Institute of Social and Preventive Medicine, Lausanne University Hospital, Lausanne CH-1011, Switzerland; 5 Health Technology Assessment Unit, University of Lausanne, Lausanne CH-1015, Switzerland; 6 Institute of Health Economics and Management, University of Lausanne, Lausanne CH-1015, Switzerland; 7 Life Course and Inequality Research Centre, Faculty of Social and Political Sciences, University of Lausanne, Lausanne CH-1015, Switzerland; 8 Alcohol Treatment Service, Lausanne University Hospital, Lausanne CH-1011, Switzerland

**Keywords:** Randomised controlled trial, Case management, Emergency department, Frequent users, Quality of life

## Abstract

**Background:**

We devised a randomised controlled trial to evaluate the effectiveness and efficiency of an intervention based on case management care for frequent emergency department users. The aim of the intervention is to reduce such patients’ emergency department use, to improve their quality of life, and to reduce costs consequent on frequent use. The intervention consists of a combination of comprehensive case management care and standard emergency care. It uses a clinical case management model that is patient-identified, patient-directed, and developed to provide high intensity services. It provides a continuum of hospital- and community-based patient services, which include clinical assessment, outreach referral, and coordination and communication with other service providers.

**Methods/Design:**

We aim to recruit, during the first year of the study, 250 patients who visit the emergency department of the University Hospital of Lausanne, Switzerland. Eligible patients will have visited the emergency department 5 or more times during the previous 12 months. Randomisation of the participants to the intervention or control groups will be computer generated and concealed. The statistician and each patient will be blinded to the patient’s allocation. Participants in the intervention group (N = 125), additionally to standard emergency care, will receive case management from a team, 1 (ambulatory care) to 3 (hospitalization) times during their stay and after 1, 3, and 5 months, at their residence, in the hospital or in the ambulatory care setting. In between the consultations provided, the patients will have the opportunity to contact, at any moment, the case management team. Participants in the control group (N = 125) will receive standard emergency care only. Data will be collected at baseline and 2, 5.5, 9, and 12 months later, including: number of emergency department visits, quality of life (EuroQOL and WHOQOL), health services use, and relevant costs. Data on feelings of discrimination and patient’s satisfaction will also be collected at the baseline and 12 months later.

**Discussion:**

Our study will help to clarify knowledge gaps regarding the positive outcomes (emergency department visits, quality of life, efficiency, and cost-utility) of an intervention based on case management care.

**Trial registration:**

ClinicalTrials.gov Identifier: NCT01934322.

## Background

Individuals attending emergency departments (ED) on a regular basis account for a disproportionally high number of all ED visits. LaCalle and Rabin [1] in their systematic review found that patients visiting an ED four or more times per year accounted for 4.5%–8% of all ED patients and 21%–28% of all ED visits. Emergency department frequent users (ED-FUs) attend the emergency department on multiple occasions; however, definitions and threshold numbers of visits vary across studies. According to Locker [2], the definition of five attendances or more per year corresponds to a non-random event and should be used to allow better comparisons between studies. ED-FUs present a higher rate of morbidity and mortality than less frequent ED users [3-7], are more at risk of drug and alcohol abuse [5,7-9], often present mental health issues [3,5,6,10], are more likely to visit for complications and exacerbations of chronic conditions [10,11], and are often homeless, uninsured, and from low socio-economic levels [3,12-14]. The majority of them believe that their complaints require immediate attention [1], and thus they constitute a significant burden on hospitals due to multiple visits and the number of problems they bring to the ED.

ED-FUs contribute significantly to ED overcrowding and extended waiting times, often due to inappropriate visits to the unit [15]. Overcrowding is detrimental to the quality of care in EDs. However, the severity of the reason for consultation at the ED is often controversial [1]. Indeed, several studies show that ED-FUs have non-emergency conditions [10,16-18] and could receive better care in settings other than an ED [19,20], which is not designed to provide continuous care to patients with non-emergency, chronic conditions. In addition, the numerous issues that ED-FUs have are not easily addressed by simply providing care alone. Appropriate and consistent medical and social services are needed for such vulnerable populations.

In response to these concerns, several institutions worldwide (e.g. in the United States, Canada, Sweden, the United Kingdom, the Netherlands, Spain, and Australia) [9,12,21-31] have introduced specific interventions for ED-FUs aimed at reducing the number of their visits, treating their medical co-morbidities, and/or addressing their social needs. Interventions vary, according to a recent systematic review of the literature by our research team that identified different types of interventions aimed at improving the management of adult ED-FUs [32] and at assessing interventions’ effectiveness. Most of the studies describe interventions referring to and/or inspired by case management (CM) [9,12,25,29-31,33].

One of the most common interventions consists of CM multidisciplinary teams composed of nurses, psychologists, and possibly physicians [27,34-39]; this approach can help address complex situations and scenarios. Team members from different professional backgrounds, such as psychiatrists and health educators might complement the team, depending on the specific CM project. Coordination and organizational care tasks are often allocated to a case manager [37] who guides patients through the care process and provides social support. Care is generally considered as a continuous integration of medical and social dimensions. It is commonly patient-centered and holistic in nature, and takes patient empowerment [27,35,36] into account. Moreover, the locus of intervention is not limited to the hospital, and often extends into the community.

CM is a highly flexible and dynamic process and mainly depends on patient needs; the order of individual steps is often not constricted. In fact, its dynamic condition emphasizes that sometimes several steps take place simultaneously, or that the case manager has to return to a previous step. Based on the literature, this can be summarized in five steps [27,38-43]: identification, assessment/reassessment, planning, implementation, and evaluation/monitoring. The Behavioural Model for Vulnerable Populations [44] provides a theoretical framework for understanding how CM might improve the care of vulnerable patients; this theoretical framework suggests that the use of health services is a function of:

• predisposition of patients (demographics, health beliefs, social structure, and childhood characteristics);

• factors that enable or impede such use (personal, family, or community resources); and

• patient need for care (perceived and evaluated health).

CM guarantees that issues in each of these domains are addressed.

Interventions aimed at improving ED-FU management have had positive outcomes: some of the interventions evaluated have been effective in reducing emergency department use [9,12,21,24,26,29,31]. Cost-reduction analyses are also promising: Wassmer anticipated reductions in cost even when partially based on modeling estimates [31]; two other studies showed the effects of clinical case management on hospital services and its cost effectiveness [12,29]. Some interventions have had positive effects on social outcomes [12], such as a significant reduction in homelessness [25,29]. A positive effect on social outcomes is essential, as the link between social problems and health has been demonstrated by many authors [45]. Finally, clinical outcomes were assessed in three studies [12,25,29]; one of them demonstrating a positive effect in reducing alcohol and drug use [12].

In the literature, interventions aimed at improving the management of ED-FUs have demonstrated several positive outcomes, but there are still some knowledge gaps:

– There is only one randomised controlled trial (RCT) showing a significant reduction in ED use by FUs compared to patients receiving standard care [29].

– The threshold for number of visits varies across the three existing RCT [22,29,30]; only one is based on the definition of five or more attendances per year, corresponding to more than known random events [29].

– Cost reductions were demonstrated in three studies [12,29,31], but only one is an RCT [29], and the other two did not contain a control group.

– Patient baseline characteristics and health-care specificities shown in 11 studies included in a systematic review by Althaus and al. [32] were only relevant within the country in which each study was conducted (the US, Sweden, Canada, Australia, and the UK).

Because of the existence of the knowledge gaps mentioned above in a topic that is of the utmost importance for patients, clinicians, and policymakers, with this trial we would like to demonstrate that by establishing locally a model of care for these patients, we can decrease the use of the health-care system, improve these patients’ quality of life, and reduce costs consequent on frequent use.

### Aims and hypotheses

The primary aim of this study is to demonstrate that an intervention on ED-FUs by a multidisciplinary mobile team (based on CM care patterns) is a more appropriate way of reducing use of the ED - through a better orientation in the health-care system - and of improving quality of life than is standard emergency care delivered by nurses and physicians, and that it will reduce associated costs.

The study tests the hypotheses that CM intervention, as compared with standard emergency care,

• reduces ED attendance through a better orientation in the health-care system;

• improves quality of life;

• is a more efficient use of health-care resources (cost vs ED attendance); and

• leads to a favourable cost-utility ratio (cost vs Quality Adjusted Life Years (QALYs)).

## Methods/design

### Study design

**Figure 1:**
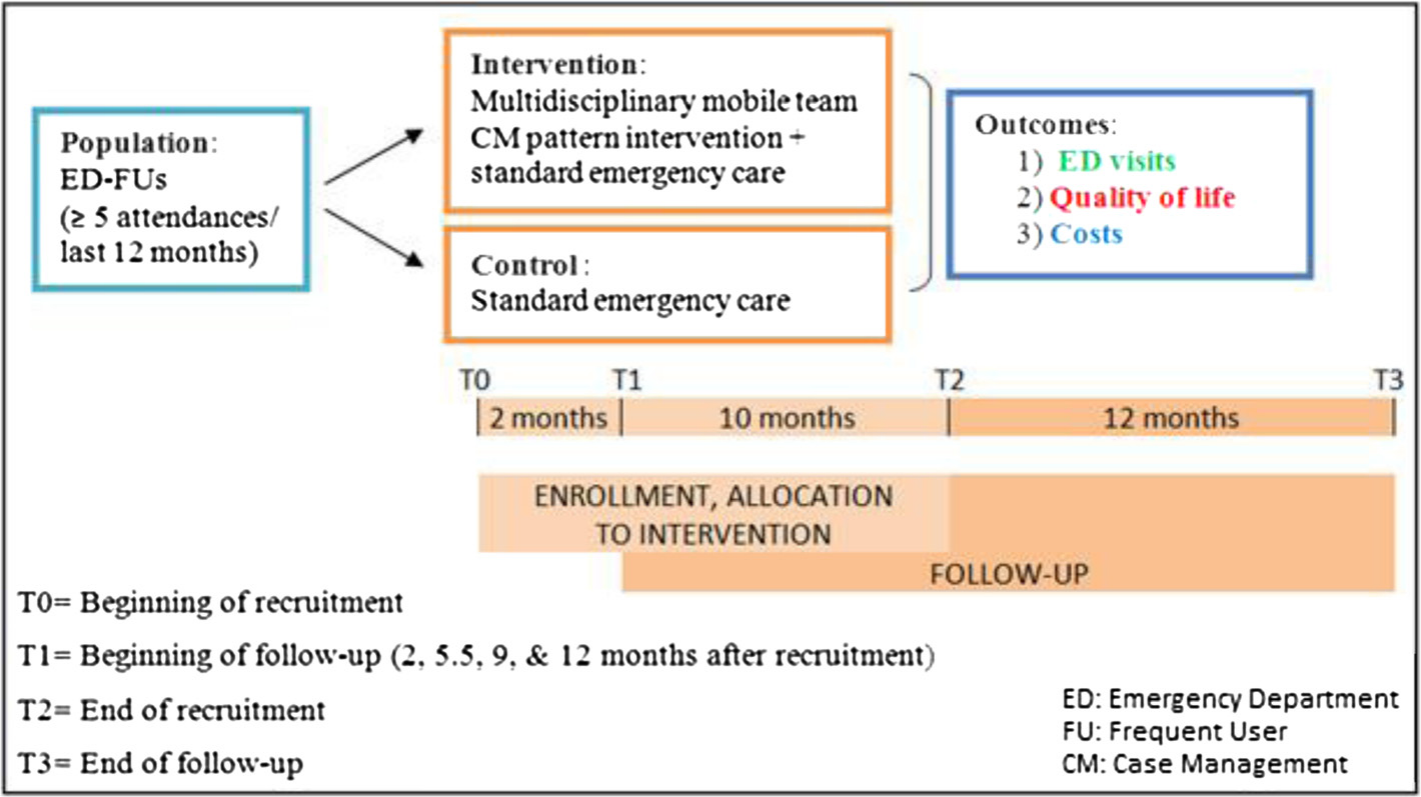
Study design: study design with inclusion and follow-up timetable.

This study is an RCT that compares comprehensive CM care associated to standard emergency care with standard emergency care alone among ED-FUs (Figure 1). The study includes a follow-up at 2, 5.5, 9, and 12 months after the first assessment.

### Setting

The study will be conducted in the Lausanne University Hospital ED. This facility is an urban public hospital serving (with other non-university hospitals) 770,000 people. It provides medical, surgical, and mental health care via 50,000 annual ED visits, and is one of the five teaching university hospitals located in Switzerland.

### Study population

#### Participants

##### Inclusion criteria

Patients presenting at an ED between T0 and T1 (12 months), will be eligible to participate, provided they are at least 18 years of age, have made five or more visits to an ED in the previous 12 months, and are capable of communicating in any of the languages spoken by the team (i.e. French, German, Italian, English, or Spanish) or through a community interpreter.

##### Exclusion criteria

Patients will not be enrolled if they cannot give informed consent or are ineligible to receive CM services (e.g. acutely confused, acutely psychotic, with dementia, or intoxicated), will not remain in Switzerland, or are not expected to survive for 18 months following enrollment. Additionally, those incarcerated, people expected to be imprisoned in the short term, and those with a family member who has already enrolled will be also excluded.

#### Flow diagram

**Figure 2:**
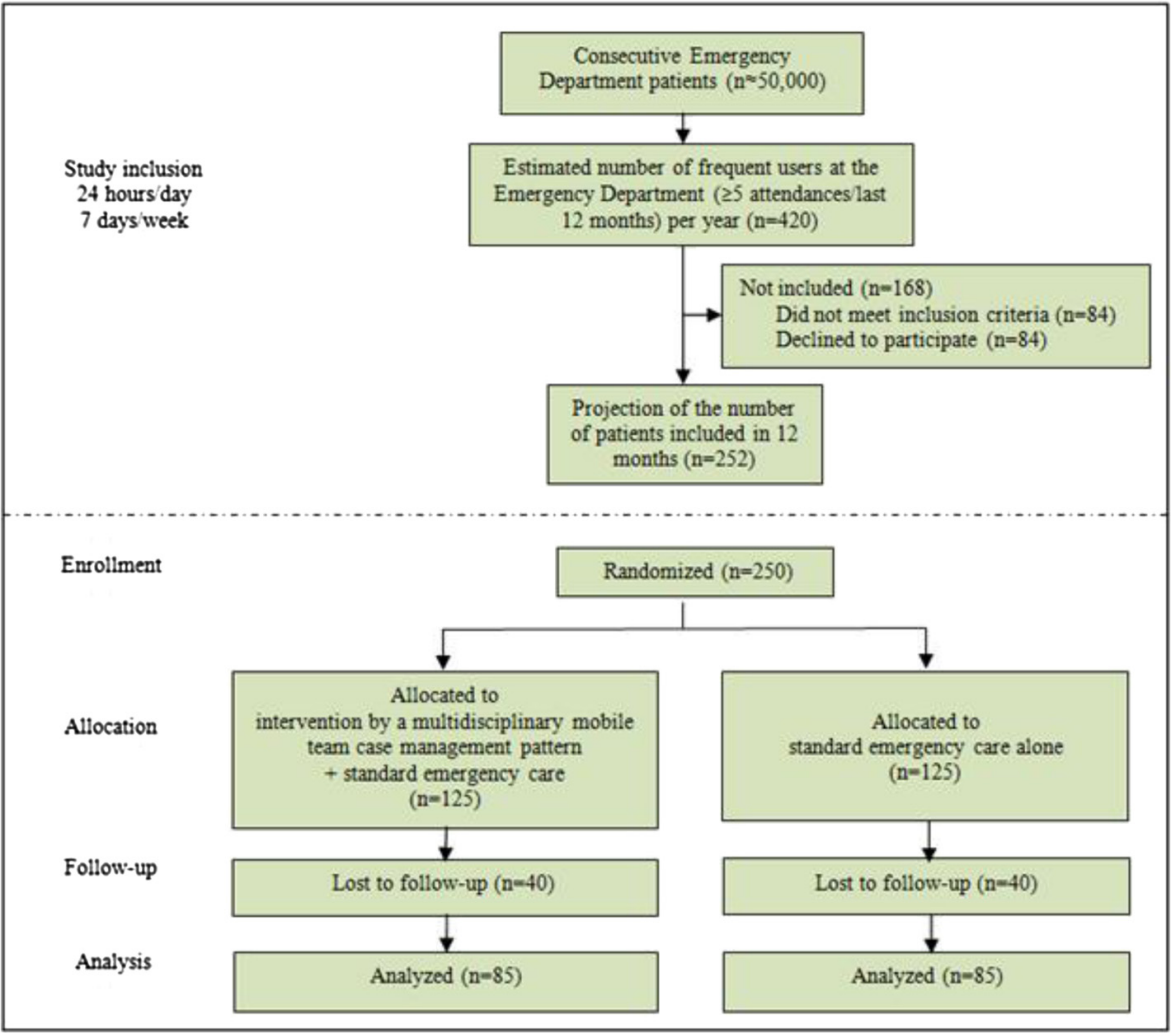
**Flow diagram: flow diagram including estimates of numbers of patients to be included based on previously published **[46,47]** and unpublished (Bodenmann P. et al., in progress) studies of ED-FUs conducted by our research group and on the power analysis conducted during study design.**

The following flow diagram (Figure 2) shows the progression through the phases of the RCT of interventions based on a multidisciplinary mobile team case management pattern, parallel to standard emergency care for ED-FUs. The numbers given in the diagram are based on the results of a recent cross-sectional study conducted in the same setting at the Lausanne University Hospital ED (Bodenmann P. et al., in progress) and on the power analysis we conducted while designing the study.

#### Recruitment

Patient recruitment will last one year (T0–›T2).

##### Frequent user identification

An automated 24-hour, seven-days-a-week detection system based on ED patient tracking software will identify all patients who will have attended the ED five times or more during the previous 12 months. A member of the CM team will approach each FU; the FU will receive written information, an oral explanation, and sufficient time to consider their opportunity to participate in the study. If the FU agrees, he or she will give his or her informal written consent. A psychologist will participate in the recruitment of the patients in order to achieve better standardization of the process and to ensure increased motivation in the participants.

If a patient is no longer in the ED, a member of the CM team will make three attempts to contact that patient by telephone within 24–72 hours of their departure from the hospital, to briefly explain the study and try to organize a meeting. If the patients has a general practitioner he/she will be alerted by telephone, email, or mail by the team member in charge of their patient. The purpose of the contact is both to inform the general practitioner and to get information from him/her.

### Allocation to conditions

#### Sequence generation

The randomisation list associating questionnaire numbers to intervention or control groups will be generated by the statistician using block randomisation prior to the start of the study. Computer-based, randomly-generated, permuted blocks of random size will assure group size balance (http://www.randomization.com). Patients will then be allocated to either group A or group B. The research team will then decide if group A or B is to be the intervention group, therefore blinding the statistician to the true allocation. The randomisation list will be held by the research team. At night and during the weekends, the CM team will be informed of ED-FU consultations via email by the ED’s staff. The CM team will contact each ED-FU the day after or on the following Monday, and if the patient agrees to participate in the study, the process of randomisation will take place in the research team office.

#### Allocation concealment mechanism

The statistician will hold the randomisation list and reveal each patient’s allocation corresponding to the questionnaire number. The allocation will be reported to the CM team by phone once baseline characteristics have been collected by the CM team. The patient will then be informed about the procedures he or she should follow, without knowing whether he or she has been assigned to the intervention or to the control group.

#### Blinding

The *research nurse*, responsible for collecting outcomes, will not be blinded to the patient’s allocation, as she had to have access to the database. The *CM team* will be blinded until randomisation. The *statistician* will be blinded to the true group until the analyses are complete. As the intervention is also provided by *ED staff* who will interact with the CM team for the intervention group patients, it is impossible to have them blinded. *Patients* will agree to take part in a study in which they will be managed by a coordinated team. *Blinding effectiveness* will be assessed by asking patients at the end of their follow-up period if they thought they were in the intervention or the control group. Since it delivers the intervention, the CM team cannot be blinded. The *data collection manager*, also responsible for quality control, will have access to all data and therefore cannot be blinded.

### Interventions

#### The multidisciplinary mobile team CM pattern intervention

The mobile team consists of four nurses practitioners. A medical supervisor (general practitioner) stages the implementation of the project, monitors the team consolidation process, and is available for medical consultations for any difficult medical conditions in patients. He has the responsibility of verifying that the intervention offered is the preferred one.

**Figure 3:**
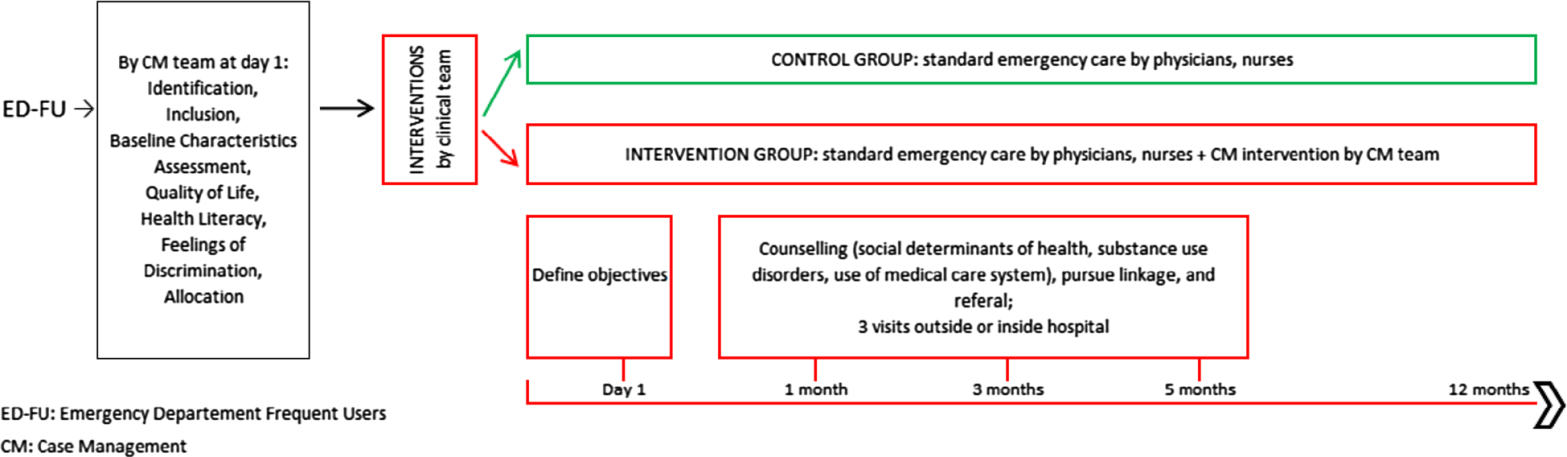
Timetable of the interventions: timetable for every ED-FU included in the study with interventions (at Day 1, 1 month, and 3 and 5 months for the intervention group).

Patients randomised to CM will receive an intervention designed to offer support for ED-FUs and the professionals who work with them inside the hospital, as well as for the community medical- and social providers who will maintain outside continuity of care:

– The CM team (four nurses) will meet the patient at the hospital or ambulatory EDs. First, they will complete an assessment of one and a half hours focussing on baseline characteristics, social determinants of health, mental and somatic diseases, risk behaviors, health-care use, and health literacy [48-50]. Second, the CM team will complete, with each patient, a questionnaire including instruments that assess quality of life (EuroQOL and WHOQOL) and feelings of discrimination.

– FUs will be seen initially by the team from one (ambulatory care) to three (hospitalization) times during their visits to the hospital and again one, three, and five months later at their home or in an ambulatory setting (Figure 3).

– In between the consultations provided, the FUs of the intervention group will have the opportunity to contact, at any moment, one of the members of the CM team in an “open-door policy perspective” with subsequent monitoring of the frequency and the content of every intervention required.

– Initial (Day 1) and follow-up interventions by the CM team (at one, three, and five months) will include counseling about social determinants of health, substance-use disorders (if relevant), and the use of medical care systems. Counseling will be based on motivational interviewing (empathy, collaboration, autonomy, and valorization), while avoiding confrontation. Each member of the CM team will have a checklist covering the proposals and advice that they have to give to every FU patient and outlining the material (flyers, addresses, etc.) that they have to provide.

– The primary goals of the interventions are to furnish specific assistance and to provide referrals for the patients:

• *If the social determinants of an ED-FU are not adequate*, the team will —

• → provide assistance in obtaining income entitlements, health insurance coverage if eligible, stable housing (e.g. shelters for the homeless), schooling for children, prevention of potential violence (i.e. conjugal and/ or against the children) in the home.

• *If there are mental disturbances*, the team will —

• → refer patients to mental health departments inside the hospital, and if necessary, to a psychiatrist, psychologist, or general practitioner (GP) out in the community.

• *If the patient presents risk behaviors (alcohol consumption, smoking, or other drug use)*, the team will —

• → refer the patient to substance abuse services and provide links to community services in order to maintain continuity of care.

• *In cases of somatic problems* (and in which the patient either has no GP or has not consulted with their GP for a long time) the team will —

• → find a new GP or make contact with the previous provider, contingent on the patient’s consent.

– Each member of the team will follow a maximum caseload of 20 patients as a case manager. We will take into account the CM team’s capacity in order to ensure consistent recruitment over time: if the program reaches capacity, particularly when the intervention group of participants is enrolled, it may become necessary to stop recruitment until clinical capacity is again available.

– The linkage to medical and social services providers inside the hospital (with the participation of the CM team in network meeting crisis interventions organized by the professionals involved in each case) will continue outside the hospital with GPs and home visits by nurses and social services. The CM team will centralize documents and facilitate communication between all care providers, ensuring ongoing community outreach in order to maintain continuity of care.

This program uses an assertive clinical CM model that is patient-identified, patient-directed, and developed to provide high intensity services. It provides a continuum of hospital- and community-based patient services that includes clinical assessment, outreach referral, and coordination and communication with other service providers. Additional components are patient education in a motivational perspective, individual counseling, crisis intervention, medical assessment, and ongoing medical care.

#### Teamwork, case conferences, continuing education

The core team (nurse practitioners, and a general practitioner) is supported by several vulnerable population experts from various hospital departments - including gynaecology-obstetrics, paediatrics, psychiatry, and ethics - who act as contact persons for their respective departments and complement the team’s interventions with their expertise on specific problems of gender, children who are minors, mental illness, and ethical concerns.

Members of the CM team will receive intensive training in motivational interviewing and cross-cultural competences, and will take specific classes in adequate referral to social assistance (e.g. income entitlements and stable housing), alcohol and drug use disorders, and home violence.

Because of the potential for difficult situations concerning many of these vulnerable patients, the members of the CM team will receive psychological support.

#### The control intervention

Patients randomised to the control group (standard care) will receive standard emergency care from physicians (resident or attending physician) and nurses, without the case manager being involved. Nevertheless, the mobile team will contact each patient in the control group, providing them with general information in the form of a flyer which will outline the existence of the mobile team, and provide relevant addresses and telephone numbers. A member of the team will then complete an assessment of one and a half hours focused on baseline characteristics, social determinants of health, mental and somatic diseases, risk behaviors, health-care use, and health literacy [48-50]. Finally, the CM team will complete, with each patient, a questionnaire including instruments that assess quality of life (EuroQOL and WHOQOL) and feelings of discrimination.

The assessment effect, if any, will be present in the control group as in the intervention group.

Concerning standard care, after the first orientation by a nurse, when an intervention is provided by a resident he or she will be systematically supervised by a chief resident. Referrals to other specialists are routinely made by residents acting as liaisons to the appropriate hospitalization sector; there is no systematic presence or involvement of nurse practitioners.

Finally, patients randomised to standard care will be eligible to receive CM services at the end of the study. In any critical situation where a patient included in the study (in the intervention or the control group) needs hospitalization, that hospitalization will occur. Nevertheless, if the patient spontaneously contacts the clinical team by means of the phone number on the flyer, he or she will be able to benefit from an intervention by the CM team (as will the intervention group), after the end of the follow-up period for the patient.

### Measurements - outcomes

**Figure 4:**
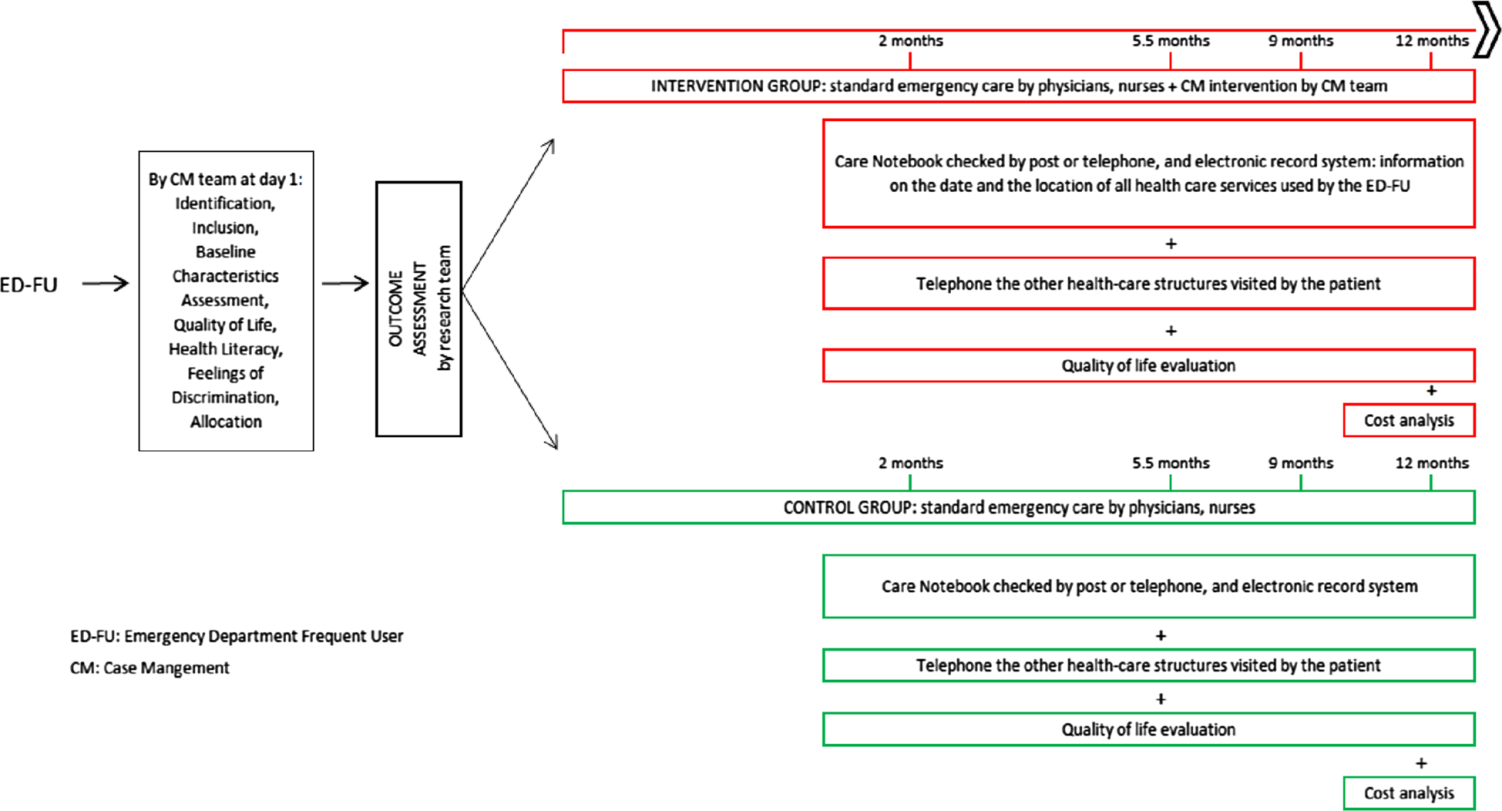
Timetable of the assessments: timetable for every ED-FU included in the study with outcome assessment (2, 5.5, 9, and 12 months for the intervention and the control group).

Questionnaires for each patient will be filled in and data will be collected at the baseline and 2, 5.5, 9, and 12 months later to assess the outcomes of the intervention. The primary outcome measurement is the number of ED visits. Secondary outcome measurements are the standardized measurements of health status via EuroQol (EQ-5D) [51,52] and WHOQOL-BREF, cost analysis based on the use of health services, and an instrument on feelings of discrimination. (Figure 4).

#### Primary outcome: number of ED visits

The primary outcome will be the number of ED visits made by FUs. This information will mainly be available via the Lausanne hospital/ambulatory electronic records system and hospital/ambulatory administrative databases covering a period of 12 months after the initial emergency department visit.

“Care Notebooks” in hands of case and control participants will also be generated from the beginning of the study. Patients will be asked to report all visits (ED visits to any hospital and all outpatient visits) in their Care Notebook during the 12 months following their first visit (Figure 3). Patients will be contacted by telephone by the nurse researcher to answer questions about their use of the health-care system and to verify that they have completed their Care Notebooks appropriately. If this is not the case, the nurse researcher will help the patient find methods and strategies for improving their reporting. If necessary, incentives would be used to help FU patients complete Care Notebooks appropriately. The quality control will be repeated at 2-, 5.5-, 9-, and (finaly) 12-month follow-ups. Confirmatory telephone calls to other hospitals, medical institutions, or private practices that the patient claims to have visited will be made by the nurse researcher, after obtaining the patient’s written permission to do so.

Moreover, the validity of FU patients’ answers could be assessed by matching their answers to our gold standard electronic records system of visits within our hospital and to the records of the participant’s health insurance provider, after having obtained relevant informed consent.

#### Secondary outcome: cost analysis

The second outcome measurement focuses on the costs induced due to the health-care resources used by the FU patients. Their health-care consumption is related to services provided by our hospital but we cannot exclude that a FU also uses services provided by other hospitals/institutions in the community (services provided outside our hospital).

1) *Concerning services provided by our hospital*, different hospital administrative databases which record all inpatient and outpatient admissions will allow us to have access to details of all healthcare used by the FU and consequently the related costs. The latter will be composed of costs related to:

a. outpatient resources induced by ED attendances,

b. inpatient resources induced by ED attendances,

c. non-ED related outpatient resources used within the hospital,

d. the ED CM multidisciplinary team intervention.

Access to the accounting analytical systems of our hospital, as well as to the outpatient invoicing department, will allow the necessary information to be collected in order to calculate costs.

2) *Concerning services used outside our hospital*, information recorded in Care Notebooks will help to identify to what extent FUs seek and use services outside our hospital’s boundaries, including whether patients use EDs of other hospitals, etc. The Care Notebook, by recording the date and the location of all visits the FU makes, will also help identify the types of services (health and/or social services) used by FUs.

The CM intervention may affect how the FUs use the health care system in general. The primary outcome of the project will allow us to identify whether the CM intervention is associated with a decrease in the number of visits to the ED of our hospital. However, it is also important to investigate to what extent the potential decrease in health-care resources used at the ED of our hospital is (or is not) associated with an increase in health-care resources used outside this specific ED. Consequently, information from the Care Notebook will help capture a substitution effect between health-care utilization at our hospital’s ED and health-care utilization external to this specific ED. Based on average unit costs, costs associated with the health care consumption outlined in the Care Notebook will be simulated.

Additionally, having obtained the patient’s informed consent we will contact their health insurer or the relevant health services provider in order to collect information on the frequency, type, and cost of health services that the participant has used during the study.

#### Secondary outcome: standardized measurement of health status via EuroQol (EQ-5D) and WHOQOL-BREF

Another secondary outcome will be the assessment of the health status of participants, as measured by the EQ-5D. This instrument is a non-disease-specific self-report of health-related quality of life. It is applicable to a wide range of health conditions and treatments, and provides a simple measurement of health that is used in clinical and economic analysis. Each respondent defines his/her own health status by combining one level (from a choice of three: “no problems”, “some problems”, “extreme problems”) from each of five dimensions: mobility; self-care; usual activities; pain/discomfort; and anxiety/depression. For any state of health reported, an EQ-5D score reflecting a health utility weight will be derived.

Quality of life will also be assessed using the WHOQOL-BREF — an instrument developed by the World Health Organization. It is a 26-item Likert scale, which focuses on four aspects of quality of life: physical health, psychological health, social relationships, and environment. It also contains two items concerning the individual’s overall satisfaction with life and general sense of personal well-being. Each response on this Likert scale is coded from 1 to 5.

To complement the assessment of the health status of participants, we will address patient satisfaction through a five-item questionnaire, validated locally.

#### Secondary outcome: feelings of discrimination

Additionally, an instrument assessing the feelings of discrimination will be filled in by each participant at the beginning of the study and 12 months later. The discrimination instrument was validated in a previous study conducted at the University Hospital of Geneva [53].

### Sample size

The sample size has been calculated to detect a between-group average reduction of two visits per year to the ED (i.e. minus four visits for the intervention group versus minus two for the control, with an expected standard deviation of four in both groups), in accordance with the results of a systematic review of the literature by Althaus et al. [32]. With a significance level of 0.05 and power of 0.9, each group should include at least 85 participants. “Given that an increased mortality rate of ED-FUs is described in the literature [54] and that, from previous observations from the CM team’s clinical activities, 30% of our patients should be refugees or undocumented migrants, we expect an increased proportion of patients lost to follow-up. We therefore voluntarily overestimated the drop-out rate for the overall population to be 30% (80/250). The total required sample size has been rounded up to 250 patients (125 in each group).

### Statistical methods

Groups will be compared from their initial allocation independently of eventual crossover (intention-to-treat analyses). The principle measurement of effect is an individual’s average reduction in visits to the ED over 12 months compared to the number of visits observed in the control group. This will be calculated using linear regression with number of visits during 12 months’ follow-up as an independent variable, and group allocation and yearly number of ED visits prior to intervention as dependent variables. Should group imbalance occur, secondary analysis will test the confounding effects by measuring the effect after adjusting for these confounding effects in the linear regression. Known determinants of frequent use are to be considered as potential confounders if, by chance due to the randomisation, we are to observe a relative difference of 20% between groups.

FUs are known to visit EDs on regular bases over a short period of time [55] (regression to the mean), so we also expect to see a decrease in the number of visits in the control group. Our analysis will measure the true effects of the intervention taking this phenomenon into consideration.

In terms of medico-economic analysis, benefits of the care management program will be evaluated by health gains expressed in Quality Adjusted Life Years (QALYs) over the 12-month period. A cost-utility analysis from the health-care provider perspective will be conducted by combining the use of two outcomes (i.e. costs and health status in terms of quality of life). A cost-utility ratio will then be calculated. A sensibility analysis will also be conducted in order to estimate the confidence interval for the cost-utility ratio. Uncertainty will be assessed by univariate and probabilistic sensitivity analysis (Monte Carlo simulation). All statistical analysis will be carried out with STATA 12.0, Statacorp, College Station, Texas, USA.

### Ethical approval

The protocol, information letters, questionnaires, and the informed consent form of the study were approved by the Human Research Ethics Committee of the Canton of Vaud, Switzerland (no 32/12). There is no expected adverse event or side effect for participants.

## Discussion

This study is coordinated with recent local research projects dedicated to assessing profiles and improving healthcare for ED-FUs, who are considered to be a highly vulnerable subgroup and a proxy for vulnerable populations in general.

At the Lausanne University Hospital ED, in 2008–2009, ED-FUs accounted for 4.4% of ED patients and 12.1% (n = 5,813) of all ED visits (n = 48,117) [46]. A retrospective chart review case–control study, conducted in this hospital between April 2008 and March 2009 by Bieler et al. [46], demonstrated that social (i.e. homelessness, institutionalization, unemployment, or dependence on government welfare) and specific medical vulnerability factors (i.e. ED primary diagnosis of substance abuse and the use of five or more clinical departments in the 12 previous months) increased the risk of ED use among 719 patients. A combination of social and medical factors was markedly associated with frequent ED use, as FUs were 10 times more likely to have three of them (of a total of eight factors; 95% CI = 5.1 to 19.6). This result is confirmed by Althaus et al. [56] in a retrospective chart review on hyperfrequent users (12 attendances or more during a year): they were 13 times more likely than non-FUs (65.5 vs 5.0%) to present three or more of the risk factors of vulnerability that Bieler et al. referred to [46] and 2.2 times more likely than FUs (62.5 vs 28.4%). Finally, unpublished, local, prospective, cross-sectional data (Bodenmann P. et al., in progress) obtained between November 2009 and June 2010 has demonstrated differences between 226 FUs and 173 infrequent users. FUs were more often younger with a mean age of 51 vs 56 in infrequent users, and the former had experienced five to 18 admissions in the previous 12 months. They cumulated vulnerabilities in terms of somatic problems, mental diseases, risk behavioral indicators, and unfavorable social determinants of health.

Taking care of a growing number of vulnerable patients requires specific interventions. A systematic review of the effectiveness of interventions targeting ED-FUs concluded that such interventions may reduce ED use and that CM, the most frequently described intervention, seemed to improve social and clinical outcomes and reduce ED costs in different studies [32]. Three studies [12,29,31], from which one RCT [29], concluded that CM could contribute to the reduction of ED use and of consequent costs, while two of these studies [12,31] found additionaly that CM could also lead to positive social outcomes. However, patterns of care that have succeeded elsewhere have to be tested in local or national settings before being introduced into a new context of care among local patients. A mixed methodology using quantitative and medico-economic analysis is needed.

Responding to the knowledge gaps in the literature [57,58] and following our local studies through different observational designs, our hypothesis is that CM leads to reduced ED use by ED-FUs through a better orientation in the health-care system, improves their quality of life, and is more cost-effective than is standard emergency care alone provided by nurses and physicians serving ED-FUs. Positive findings would constitute a strong incentive to replicate these studies on a larger scale, in a multicenter study with more extensive follow-up procedures. Positive findings would also suggest that specific populations need specific care, and would have major implications for healthcare quality and costs. Finally, the total number of ED visits in Switzerland is around 1.3 million per year [59] and has been steadily growing. If our intervention results in a reduction in the number of ED visits, the impact at the national level could be significant.

## Abbreviations

RCT: Randomised controlled trial; ED: Emergency department; FU: Frequent user; ED-FU: Emergency department frequent user; CM: Case management; GP: General practitioner.

## Competing interests

The authors declare that they have no competing interests.

## Authors’ contributions

PB, VSV, and OR wrote the manuscript. All authors critically reviewed the manuscript for important intellectual content. The study design and research proposal were mainly developed by PB and JBD. OH, BB, JBW, KI, KM, and SB made substantial contributions to the conception and design of the study. PB, JBW, and KM performed the power analysis. The intervention was developed by PB, OH, and JBD. All authors agree to be accountable for all aspects of the work in ensuring that questions related to the accuracy or integrity of any part of the work are appropriately investigated and resolved. All authors have read and approved the final manuscript.

## Pre-publication history

The pre-publication history for this paper can be accessed here:

http://www.biomedcentral.com/1472-6963/14/264/prepub
